# Impairments in Background and Event-Related Alpha-Band Oscillatory Activity in Patients with Schizophrenia

**DOI:** 10.1371/journal.pone.0091720

**Published:** 2014-03-19

**Authors:** Ilana Y. Abeles, Manuel Gomez-Ramirez

**Affiliations:** 1 Program in Cognitive Neuroscience, Department of Psychology, The City College of the City University of New York, New York, New York, United States of America; 2 Program in Cognitive Neuroscience and Schizophrenia, Nathan S. Kline Institute for Psychiatric Research, Orangeburg, New York, United States of America; 3 The Zanvyl Krieger Mind Brain Institute, The Johns Hopkins University, Baltimore, Maryland, United States of America; Osaka University Graduate School of Medicine, Japan

## Abstract

Studies show that patients with schizophrenia exhibit impaired responses to sensory stimuli, especially at the early stages of neural processing. In particular, patients’ alpha-band (8–14 Hz) event-related desynchronization (ERD) and visual P1 event-related potential (ERP) component tend to be significantly reduced, with P1 ERP deficits greater for visual stimuli biased towards the magnocellular system. In healthy controls, studies show that pre-stimulus alpha (background alpha) plays a pivotal role in sensory processing and behavior, largely by shaping the neural responses to incoming stimuli. Here, we address whether patients’ ERD and P1 deficits stem from impairments in pre-stimulus alpha mechanisms. To address this question we recorded electrophysiological activity in patients with schizophrenia and healthy controls while they engaged in a visual discrimination task with low, medium, and high contrast stimuli. The results revealed a significant decrease in patients’ ERDs, which was largely driven by reductions in pre-stimulus alpha. These reductions were most prominent in right-hemispheric areas. We also observed a systematic relationship between pre-stimulus alpha and the P1 component across different contrast levels. However, this relationship was only observed in healthy controls. Taken together, these findings highlight a substantial anomaly in patients’ amplitude-based alpha background activity over visual areas. The results provide further support that pre-stimulus alpha activity plays an active role in perception by modulating the neural responses to incoming sensory inputs, a mechanism that seems to be compromised in schizophrenia.

## Introduction

Neural activity can exhibit temporally structured patterns in response to external sensory inputs, motor actions, cognitive states, but also to the absence of sensory stimulation during resting state conditions [1], [2], [3], [4], [5], [6]. These temporally structured signals are believed to play fundamental roles in perception by controlling the excitability state of a local neural population [6], [7], [8], [9], [10], [11] and mediating the interplay between areas in a functional neural network [12], [13], [14], [15]. In particular, studies show that alpha-band oscillations (8–14 Hz) regulate stimulus-related responses [10], [16], [17], [18], [19], [20], [21], predict subjects’ behavior [18], [22], [23], [24], [25], [26], [27], and abnormal patterns in alpha activity have been characterized in several psychiatric and neurological disorders [28], [29], [30].

Alpha rhythms can be classified based on their frequency speed (low-band vs. high-band alpha frequency; see [31] for details) but also on their event driven responses [32], [33]. For instance, phasic-alpha oscillations are alpha signals in response to a sensory stimulus or an endogenous cognitive event (i.e. event-related) that occur over relatively short timescales (∼100–1000 ms), and tend to display retinotopic-specific topographies [34], [35]. An example is the event-related desynchronization (ERD), which is a reduction in post-stimulus alpha amplitude as compared to the pre-stimulus period that is thought to reflect the release of inhibition in areas encoding sensory inputs [36]. Another example is the alpha ‘distracter suppression’ effect, which is an increase in post-stimulus alpha usually following the ERD that is thought to reflect neural suppression in the regions that encode irrelevant stimuli [37].

In addition to these event-related alpha oscillations, studies have investigated alpha changes unfolding over relatively longer periods of time that are not necessarily locked to sensory stimulation. This form of alpha, referred to as background alpha (sometimes tonic alpha or pre-stimulus alpha), is often computed in the period preceding a cue or target stimulus, and as such, it is not strictly associated with the presentation of a sensory stimulus (unlike the ERD) or an active deployment of attention (unlike the alpha ‘distracter suppression’ effect). Rather, background alpha seems to represent a general state of sustained attention and task engagement, which usually displays a different topographical distribution, relative to phasic-alpha, that is concentrated over central posterior regions [27], [33]. Further, background alpha has been shown to modulate broadband evoked responses to incoming visual and auditory sensory inputs [16], [19], [20], [21], [38], [39], [40], [41]. Moreover, clinical studies have shown a substantial reduction in schizophrenia patients’ background alpha during resting state conditions with eyes opened or closed while patients were taking different types of medication [42], [43], [44], [45].

**Figure 1:**
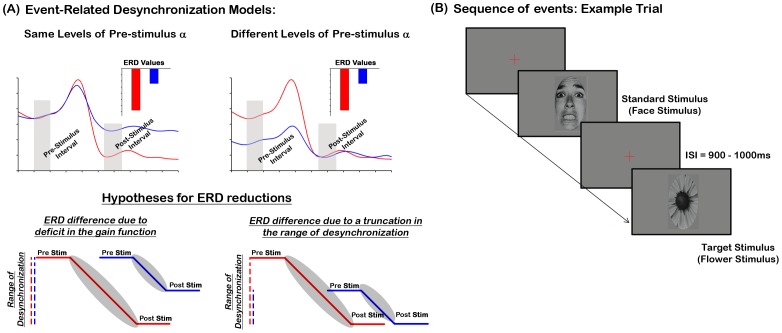
Putative models of dysfunctional ERD and the experimental design sequence of events. (A) This figure illustrates putative models explaining deficits in patients’ ERD. The top graphs are artificially constructed alpha-band waveforms between two groups (e.g. red trace = controls, blue trace = patients), where the ERD is relatively lower for one group (blue trace) compared to the other (red trace, averaged ERD values shown in the inset). The lower panels indicate two hypotheses explaining the ERD reductions. These ERD differences can arise from deficits in gain mechanisms (caused by post-stimulus alpha impairments, lower left-graph) or deficits in the range over which alpha can desynchronize (caused by low levels of pre-stimulus alpha, lower right-graph). The vertical hatched lines indicate the level of pre-stimulus alpha. The gray-oval shapes encapsulating the slanted lines highlight the amount (i.e. gain) of desynchronization. Note that the blue slanted lines inside the two oval shapes are of similar length but have dissimilar ranges for desynchronizing alpha (left graph>right graph). (B) This figure illustrates a typical trial in the experiment. Facial emotion stimuli were presented 90% of the time at three different luminance contrasts and a target flower was presented at only one high contrast 10% of the time. Participants were instructed to press a button to the target flower and ignore all other stimuli.

Anomalies in alpha-band activity of schizophrenia patients have also been found in stimulus-related responses, with the majority of studies reporting a significant decrease in patients’ alpha ERD [46], [47], [48], [49], [50], [51]. The etiology of this reduction is unknown. Yet, because the ERD is based on both pre- and post-stimulus alpha (see equation 1), ERD deficits may stem from abnormalities in background alpha or gain-related desynchronization alpha-band mechanisms. In particular, we posit that ERD reductions that are accompanied by normal levels of pre-stimulus alpha suggest that diminished ERDs are due to deficits in the amount of alpha desynchronization (i.e. gain desynchronization mechanisms; Figure 1a upper and lower left-most graphs). However, ERD reductions that are accompanied by lower levels of pre-stimulus alpha and normal levels of post-stimulus alpha, may be interpreted as impairments in the range over which alpha is able to desynchronize (i.e. a narrower window for desynchronizing activity; see Figure 1a upper and lower right-most graphs). Certainly, concurrent deficits in both types of alpha mechanisms are also possible.

In addition to impaired responses in post-stimulus alpha activity, patients with schizophrenia also manifest acute deficits in broadband responses to sensory stimuli. Particularly, studies have shown decreases in patients’ visual P1 event-related potential (ERP) component [52], [53], [54], [55], [56], with the deficit being larger for stimuli biased towards the magnocellular pathway [53], [57], [58], [59], [60]. The leading tenet indicates that this impairment in magnocellular processing is a major factor underlying patients’ sensory and cognitive neural deficits [56], [58]. We contend that reductions in patients’ background alpha-band activity [42], [45], [61] are also major contributors to their deficits in stimulus-related responses. Indeed, a collection of studies have shown that background (or pre-stimulus) alpha activity modulates stimulus evoked responses such as the P1, N1 and P300 ERP components of healthy controls [16], [17], [19], [21], [38], [39], [40], [41], and intracranial recordings in non-human primates show that the baseline oscillatory activity of a local neural population can have a significant impact on stimulus-related activity [6], [7], [62]. Thus, it is likely that patients’ background alpha activity has strong bearing on their post-stimulus sensory response.

The goal of this study was to investigate the relationship between background alpha activity, the ERD and visual P1 stimulus-related responses in patients with schizophrenia. While many studies have reported deficits in patients’ background alpha, these impairments have predominately been assessed during resting state conditions. Here, we investigate background alpha-band activity during an active task, but more importantly, we query its role in regulating patients’ sensory processing, including stimuli biased towards the magnocellular pathway.

## Materials and Methods

### Ethics Statement

The Nathan Kline Institute and Rockland County Department of Mental Health Institutional Review Boards (IRB) approved the study, and all participants provided written informed consent in accordance with the principles of the Declaration of Helsinki. Consent was obtained by research personnel who were trained and demonstrated competency in understanding the ethical obligations of the informed consent process. The person obtaining informed consent discussed the study with the participant in detail, providing an explanation of the study, its risks, benefits, procedures, what would be required of the participant during the study, and assessed the participant’s comprehension of the material. Participants were included in the study if they had full capacity to consent. Participants were not disadvantaged in any way by not participating in the study.

### Participants

Participants were 28 patients meeting DSM-IV criteria for schizophrenia or schizoaffective disorder and 25 age-matched healthy volunteers. Patients were recruited from inpatient and outpatient facilities associated with the Nathan Kline Institute for Psychiatric Research (NKI). Diagnoses were obtained using the Structured Clinical Interview for DSM-IV (SCID) and other available clinical information. Healthy volunteers with a history of SCID-defined Axis I psychiatric disorder were excluded. In addition, participants with history of any neurological or ophthalmologic disorder that might affect performance, substance dependence within the last 6 months, or abuse within the last month were also excluded. All participants had 20/32 or better corrected visual acuity on the Logarithmic Visual Acuity Chart (Precision Vision, LaSalle, IL), and received a moderate fee for their participation. For analyses of the neurophysiology data we excluded a subset of participants due to noisy ERP data (4 controls and 7 patients; see below). Thus, there were 21 participants per subject-group in all analyses. Clinical and demographic information for these participants are included in Table 1. All patients were receiving antipsychotic medication at the time of testing. Chlorpromazine (CPZ) equivalents were calculated using conversion factors described previously [63]. Five patients were on combined therapeutic medication (atypical+typical) and sixteen were on atypical antipsychotics.

**Table 1:** Participant Characteristics.

Characteristic	Controls (n = 21)	Patients (n = 21)	P-value^a^
**Diagnosis**			
Schizophrenia		18	
Schizoaffective Disorder		3	
**Age (years)**	38.4±12.8	42.1±10.9	0.3
**Gender (M/F)**	15/6	20/1	0.09^b^
**Chlorpromazine daily equivalent (mg)**		811.4±1.6	
**Antipsychotics**			
Atypical Only		16	
Typical Only		0	
Both		5	
**Parental socioeconomic status**	41.4±18.4 (n = 20)	38.4±15 (n = 14)	0.6
**BPRS total score**		39.1±6.4 (n = 18)	
**SANS total score (not including global scores)**		38.8±13.2	
**Duration of illness (years)**		15.9±9.2	
**IQ (Quick test)**	109.2±11.9 (n = 20)	95.6±8.8	<.001

Values are mean ± SD.

Numbers of subjects per group are noted when there is missing data. Socioeconomic status was measured by the 4-factor Hollingshead Scale. M, male; F, female; BPRS, Brief Psychiatric Rating Scale; SANS, Schedule for Assessment of Negative Symptoms; IQ, Intelligence Quotient. (^a^ *P* values from t tests. ^b^ *P* value from Chi-Square test).

### Stimuli

Stimuli consisted of faces depicting fearful, happy, sad or neutral expressions from 11 different individuals extracted from the Ekman and Friesen database [64]. An oval mask was placed around the facial image to reduce cues such as gender or age. The contrast value of each face was altered to 2, 8, and 57% root-mean-square contrast using the gray levels within the oval aperture. Stimuli were presented centrally on a Phillips CRT monitor located 114 cm from participants with the mean luminance held constant. In Fourier space, the mean luminance is the zero spatial frequency. By setting that particular point equal across all images, it has the effect of setting mean luminance to the same level. Major and minor axes of stimuli subtended 5°×7° of visual angle. A flower stimulus enclosed in the same sized oval as the facial images served as the target stimulus and was presented at 57% contrast with the same mean luminance as the facial images.

### Procedure

A typical sequence of events is illustrated in Figure 1b. Standard and target stimuli were randomly intermixed and presented 90% and 10% of the time, respectively. Stimuli were presented for 500 ms with an inter-stimulus-interval (ISI) uniformly jittered between 900 and 1100 ms. Participants were required to press a button to the flower image and ignore all other stimuli. This allowed us to probe the physiological data related to face stimuli free of any overt manual response. All participants completed 30 blocks, and each block was composed of 120 trials. Analysis of face stimuli was collapsed across emotion conditions since our main goal was to study the effects of pre-stimulus alpha on basic sensory processing.

### Data Acquisition and Processing

High-density continuous EEG was acquired from 64 surface electrodes arranged geodesically, using the BioSemi Active II system (BioSemi, Amsterdam, The Netherlands). Data were digitized online at 512 Hz, and recorded relative to a common-reference during acquisition. Data were re-referenced offline to the average of all electrodes. Only EEG data associated with the standard stimuli were analyzed.

Analyses of the data were carried out using in-house analysis scripts in Matlab (Natick, Massachusetts). Data were bandpass filtered (.05–110 Hz) using a standard Butterworth filter, and downsampled to 256 Hz. EEG epochs were derived from −500 to 700 ms relative to the onset of the standard stimulus. A baseline measure defined as −100 to 0 ms was applied to each individual epoch before performing the wavelet time-frequency decomposition method. For each epoch, individual ‘noisy’ time points in a channel were splined-interpolated if the average signal from that channel exceeded ±2 standard deviations from the average of the 3 most-neighboring electrodes. However, if more than 25% of the channel’s data points exceeded this threshold, the whole epoch was reconstructed by spline-interpolating the data from the 3 most-neighboring electrodes. This was done by implementing these steps: (1) averaging the data from the neighboring electrodes, (2) randomly removing 25% of the points, and (3) applying the spline-interpolation method. Only neighboring channels that were below the artifact threshold were used. On average, less than 1% of trials were interpolated in each group. These values were not statistically significant across groups (t(40) = 1.63, p = 0.11). An artifact rejection criterion of ±125 microvolts (μV) was used at all other electrode sites to exclude periods of high EMG and other noise-transients. Trials with eye blinks and large eye movements were rejected offline. This was defined as continuous deviations of at least ±10 μV for more than ∼97 ms on both eye channels relative to a 10 ms baseline period. We excluded a subset of participants (4 controls and 7 patients) because their percentage of accepted trials was below 70%.

### Data Analysis in the Time-frequency Domain

Instantaneous oscillatory amplitude was characterized by applying a Morlet wavelet decomposition method on single trials [65], and then rectifying the wavelet convolved signal. For each condition, the wavelet-transformed trials were then averaged, thus providing a combined measure of phase-locked and not strictly phase-locked activity unfolded in time. For each accepted epoch, the wavelet decomposition was computed from 8 to 14 Hz (the alpha-band). The Morlet wavelet was applied with a scaling window of 7 (i.e. the kappa parameter).

**Figure 2:**
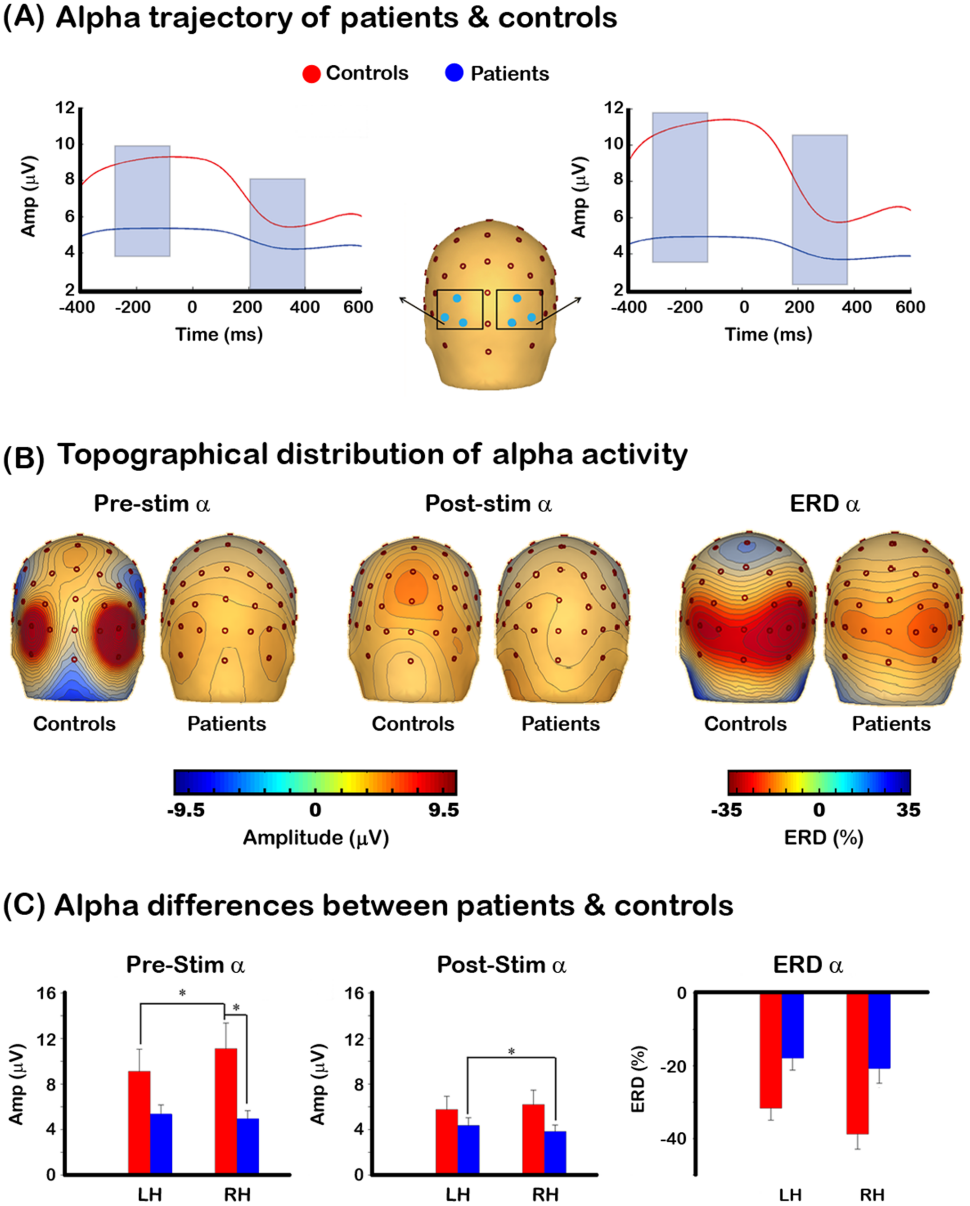
Alpha-band activity in patients and healthy controls. (A) Instantaneous alpha-band amplitude, collapsed across contrast conditions, in patients and controls. The traces correspond to the average activity between the three electrode sites illustrated in the head model (B). This figure shows the topographical distributions for each time period of interest in controls and patients. The data show a focal bilateral distribution in pre-stimulus alpha for the controls and patients (patients’ activity is substantially reduced). The post-stimulus alpha topography shows a weak parieto-occipital central distribution for the controls. The ERD topography in both groups shows a bilateral distribution with higher activity over the right hemisphere. (C) This figure illustrates alpha differences between groups. The data shows greater activity in controls vs. patients in every measure. LH = left hemisphere, RH = right hemisphere. Red traces and bar graphs correspond to activity of healthy controls, while blue traces and bar graphs correspond to activity of patients.

Statistical analyses of the instantaneous alpha-power were carried out on the pre-stimulus and post-stimulus time periods of the epoch. Statistical testing was also performed on the ERD, which was derived using equation (1):
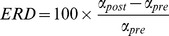
(1)


where *α_Pre_* and *α_Post_* equal the mean activity in alpha during the pre- and post-stimulus period, respectively [66], [67]. Pre-stimulus activity was computed by averaging activity from −300 to −100 ms, while post-stimulus activity was derived by averaging activity between 200–400 ms. Separate averages were made for each hemisphere by averaging across a cluster of symmetrical electrodes in the left (O1, PO3 and PO7) and right (O2, PO4 and PO8) hemispheres. These clusters were chosen based on the maximal activations observed in the averaged topography between patients and controls (see Figure 2B). Note that while some studies report maximal background alpha over centro-parietal regions [33], our data shows maximal activations over these posterior bilateral electrodes.

Separate mixed-models Analysis of Variance (ANOVA) were conducted for the pre-stimulus, post-stimulus, and ERD alpha periods. Each ANOVA had a between-subjects factor of Group (patient vs. control) and two within-subjects factors of Contrast (2%, 8%, and 57%) and Hemisphere (left vs. right). Post-hoc planned comparison t-tests were conducted when appropriate. Correlation analyses between the CPZ equivalents, symptoms, and the alpha-band measures were computed to examine the relationship between the symptomatology and neurophysiology.

### Relationship between Pre-stimulus Alpha-power, ERD and P1 ERP Component

Separate analyses were performed to assess whether changes in pre-stimulus alpha lead to modulations in the P1 ERP component and alpha ERD. For each accepted trial, in each participant, the amplitude of the P1 and ERD were sorted with respect to the pre-stimulus alpha amplitude. Pre and post-stimulus alpha outliers consisting of values outside ±2 standard deviations from the mean were removed. The P1 and ERD values were then divided into 20 bins and the values in each bin were averaged to smooth out noisy data. Bin 1 and 20 consisted of P1 or ERD values associated with the 5% smallest and largest pre-stimulus alpha values, respectively. Given that stimuli with different contrasts modulate P1 amplitudes nonlinearly [53], we performed the analysis separately on each contrast condition. The temporal windows for the P1 component were the following: 85–125 ms (57% contrast), 105–145 ms (8% contrast) and 125–165 ms (2% contrast). These values correspond to the maximal response of the group-average during the P1 timeframe. For the ERD, we collapsed across contrast values. The temporal window for the ERD was 200–400 ms post-stimulus onset. The temporal window for pre-stimulus alpha was −300 to −100 ms. The same clusters of electrodes as above were used (i.e. O1, PO3, PO7, O2, PO4 and PO8). The data across the left and right hemispheres were collapsed since no differences were observed in these analyses. The sorted data were submitted to regression analyses for statistical testing.

## Results

### Alpha-band Effects between Groups

Alpha-band waveform trajectories, collapsed across contrast conditions, are plotted in Figure 2A for both patient and control groups. Figure 2B illustrates the pre-, post- and ERD alpha voltage topographies in both groups. The bar graphs in Figure 2C show the mean amplitude in the pre- and post-stimulus time windows and the ERD for both groups.

#### Pre-stimulus activity

The mixed-model ANOVA conducted on the pre-stimulus time period revealed a main effect of Group (F_1,40_ = 5.0, *p = .03*), with patients having lower pre-stimulus alpha activity relative to controls (see Figure 2C left panel). The ANOVA also showed a significant Group x Hemisphere interaction (F_1,40_ = 8.2, *p = .007*). Follow-up independent samples t-tests revealed that this effect was largely driven by a greater alpha amplitude difference over the right vs. left hemisphere in controls relative to patients (t_40_ = −2.6, *p = .016*; Figure 2C, left panel). The size of this effect, measured by Cohen’s d [68], was 0.81, and the statistical power (β) was 97%.

Paired samples t-tests showed that controls had greater alpha power in the right compared to the left hemisphere (t_20_ = 2.45, *p = .024*), whereas patients showed a trend for greater alpha power in the left vs. right hemisphere (t_20_ = −1.9, p = .07) (Figure 2C, left panel). As expected, there was no main effect of contrast in this period. No other significant effects were observed in this period.

#### Post-stimulus activity

There were no significant main effects of Group (F_1,40_ = 1.95, p = .17) or Hemisphere (F_1,40_ = .067, p = .8). However, the ANOVA revealed a significant Group x Hemisphere interaction (F_1,40_ = 5.79, *p = .02*). Follow-up paired-samples t-tests revealed that this interaction was driven by patients having greater alpha-band amplitude in the left vs. right hemisphere (t_20_ = −3.1, *p = .006;* Figure 2C middle panel). No other significant effects were observed in this period.

#### Alpha event-related desynchronization (ERD)

There was a significant main effect of Group (F_1,40_ = 9.58, *p = .004*; Figure 2C, right panel) driven by controls exhibiting greater ERD relative to patients. There was also a significant main effect of Hemisphere (F_2,39_ = 15.8, *p = 9.42*×*10^−6^*), such that greater ERD was observed over the right vs. left hemisphere. No significant interactions were observed.

We examined whether the Group main effect in the ERD was a result of patients’ inability to produce the ERD effect itself. This was done by computing a one-sample t-test (against zero) on the ERD of patients, averaged across left and right hemispheres. The results revealed a significant effect (t_20_ = 5.2, *p = 2.17*×*10^−5^*), demonstrating that patients exhibit an ERD. In summary, these findings support the hypothesis that patients’ ERD deficits arise from lower levels in background alpha, which in turn truncate the range over which alpha can desynchronize before reaching floor levels (Figure 1A right side graphs).

**Figure 3:**
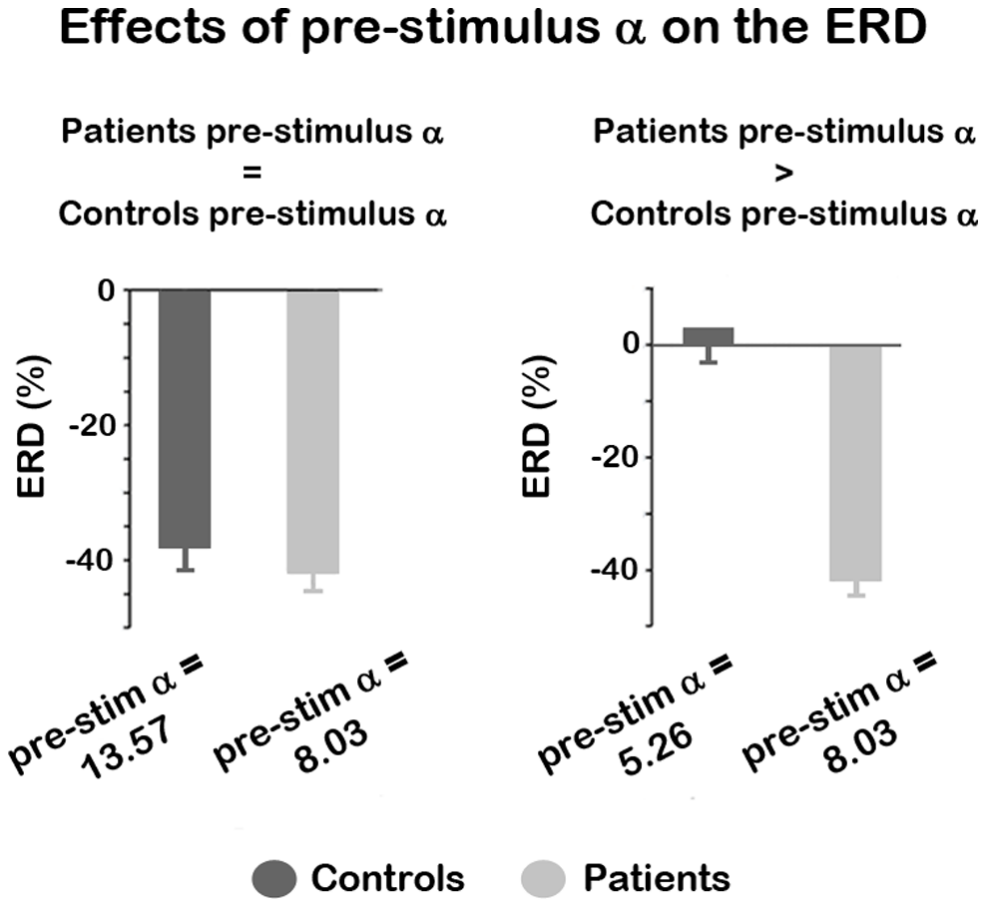
Different levels of background alpha lead to ERD modulations across groups. The left graph shows that when pre-stimulus alpha levels are equated across the groups, the ERD values are not significantly different from each other. The right graph shows that the ERD of controls is substantially lower than those of patients when controls have significantly lower pre-stimulus alpha.

To further examine whether patients’ ERD deficits arise from lower levels of background alpha, we computed an additional analysis where we equated levels of pre-stimulus alpha across both groups. This was done by sorting each single-trial ERD value in both groups with respect to pre-stimulus alpha, and grouping these values into 20 different bins. We selected the ERD values associated with the top 30% pre-stimulus alpha values in patients (i.e. top 6 bins) and compared them to the ERDs of controls whose top pre-stimulus alpha bin values were not significantly different from the top 30% of patients’ pre-stimulus alpha. This was done by removing the top binned pre-stimulus alpha values of controls until the values between the groups were not significantly different from each other. We then computed independent samples t-tests on the associated ERDs to test for statistical significance. The t-test revealed no significant differences in ERDs across groups (t_39_ = −0.90; p = .19; see left graph Figure 3). These results indicate that when pre-stimulus alpha activity is equated across groups, the ERD is no longer significantly different from each other, further supporting the hypothesis that ERD differences between patients and controls are largely caused by reductions in patients’ background alpha activity.

We performed an additional analysis in which we compared the ERD values of patients vs. controls when the background alpha values of patients were significantly larger than those of controls. This analysis was performed to test whether deficits in patients’ ERD are solely due to a truncation in the range of pre-stimulus alpha levels, or whether they also arise from impairments in gain mechanisms. The assumption is that if patients have higher pre-stimulus alpha levels than controls, they should have a higher range over which alpha can desynchronize, or conversely controls have a lower range over which their alpha can desynchronize. This analysis was performed using a similar method as above. Specifically, each single-trial ERD value in both groups was sorted with respect to pre-stimulus alpha and grouped into 20 different bins. We selected the ERD values associated with the top 30% pre-stimulus alpha values in patients and compared them to the ERDs of controls whose top pre-stimulus alpha bin values were significantly lower than the top 30% of patients’ pre-stimulus alpha. This was done by removing the top binned pre-stimulus alpha values of controls until the values of patients were significantly greater than controls. We then computed independent samples t-tests on the ERDs to test for statistical significance. These data are presented in the right graph of Figure 3, which show that when pre-stimulus alpha activity is larger in patients (t_39_ = 2.19, *p = 0.017*), their ERD values are also greater as compared to controls (t_39_ = 11.64, *p = 1.46*×*10^−14^*).

### Alpha-band Activity Across Contrast Levels

**Figure 4:**
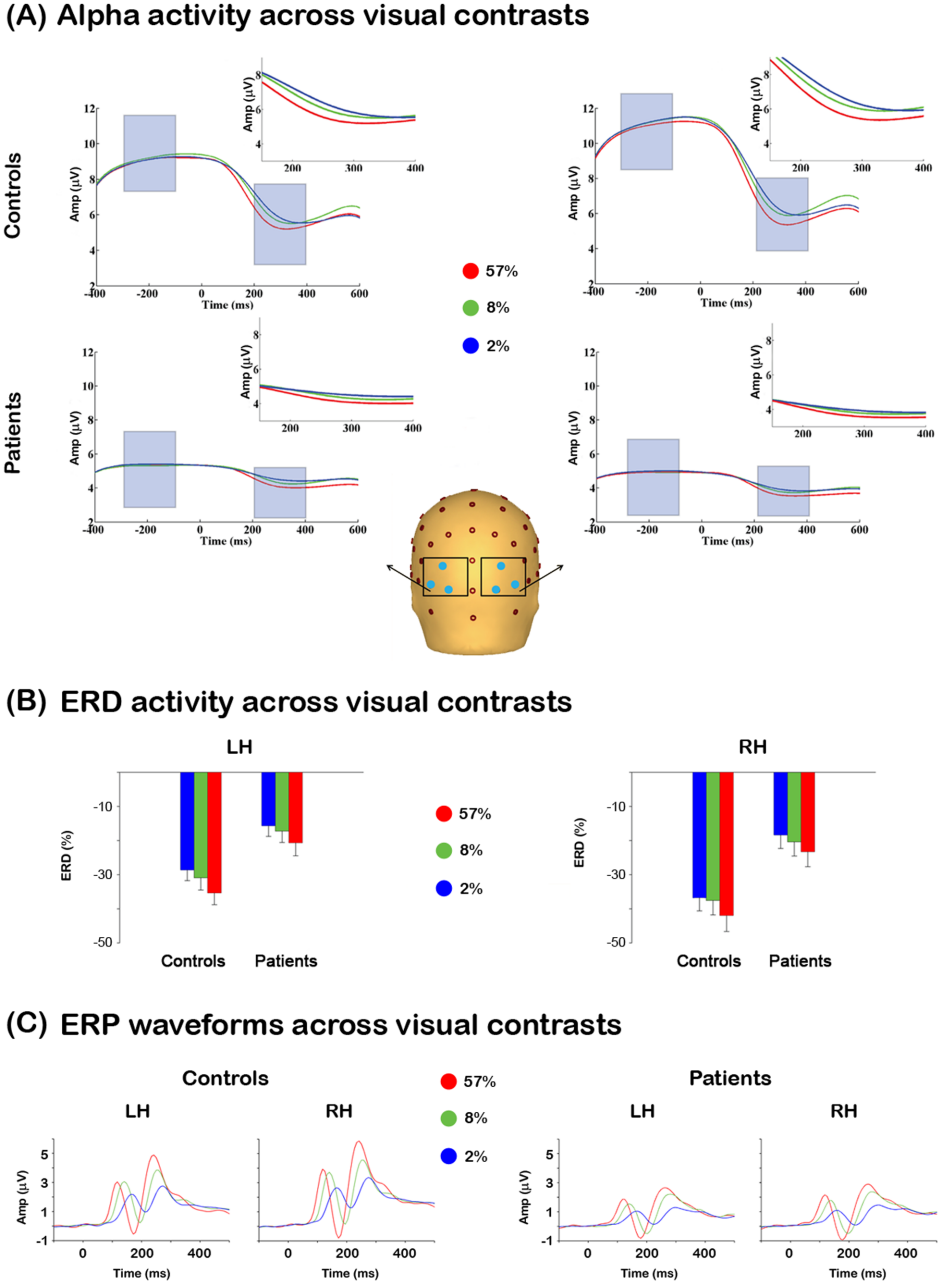
Alpha-band and P1 activity across visual contrasts conditions. (A) This figure illustrates the instantaneous alpha-band amplitude across different contrast conditions in patients and controls. (B) ERD differences between groups across visual contrasts. The figure shows an approximate linear relationship between visual contrasts and ERD, whereby higher contrast stimuli elicit higher ERD responses in both groups. (C) ERP responses between groups across visual contrasts. The figure shows the ERP response to the different visual contrast conditions. The graphs display the typical P1 nonlinear response across visual contrast in controls and patients. LH = left hemisphere, RH = right hemisphere.

Alpha-band waveform trajectories for each group and contrast condition are plotted in Figure 4A. The topographical distribution of alpha activity across contrast conditions was similar between the groups (data not shown). As expected, in the pre-stimulus time period, the main effect of Contrast was not significant (F_2,39_ = .62, p = .54), nor were the Group x Contrast (F_2,39_ = .68, p = .51), or Hemisphere x Contrast (F_2,39_ = 1.3, p = .28) interactions (Figure 4A). However, for the post-stimulus period, there was a significant main effect of Contrast (F_2,39_ = 15.2, *p = 1.31*×*10^5^*), whereby greater increases in contrast led to greater decreases in alpha-amplitude (see insets Figure 4A). Similarly, the ANOVA computed on the ERD showed a significant main effect of Contrast (F_2,39_ = 14.9, *p = 1.56*×*10^−5^*), with greater contrast resulting in a greater ERD (Figure 4B). No other significant differences related to contrast were observed. Figure 4C shows the waveforms of the ERP components, which replicate previous studies [58].

### Relationship between Pre-stimulus Alpha and Stimulus Processing

**Figure 5:**
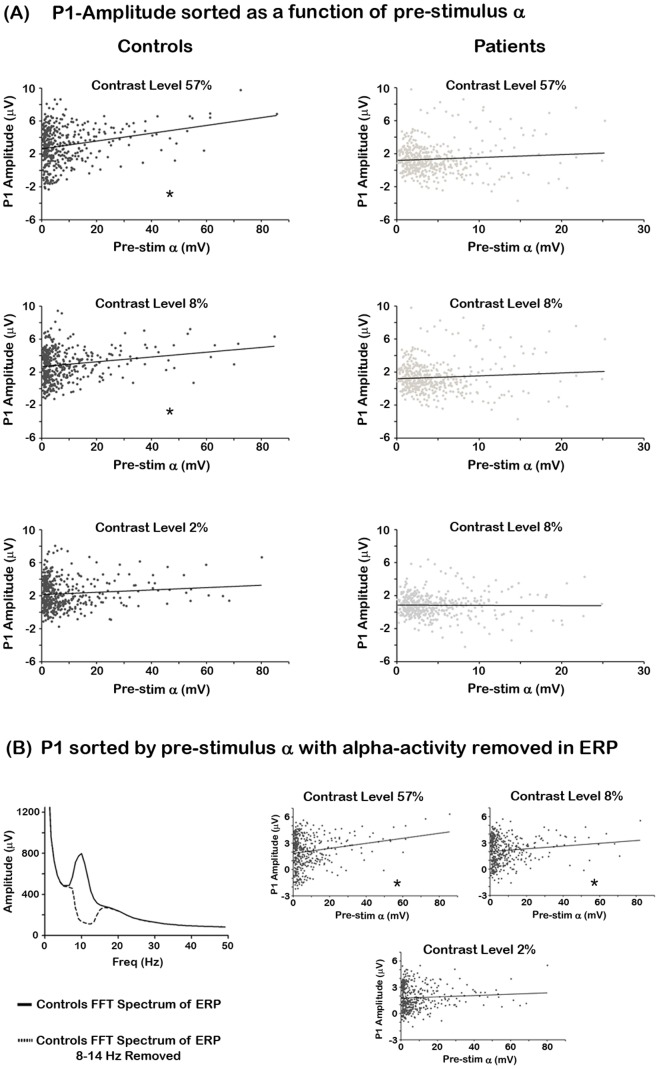
Relationship between the P1 amplitude and pre-stimulus alpha. (A) Amplitude values of the P1 ERP component plotted as a function of binned pre-stimulus alpha amplitude on a single trial level. (B) This figure shows the relationship between the P1 amplitude and pre-stimulus alpha after removing post-stimulus alpha-band activity. The graph on the left shows the FFT spectrum of the ERP with alpha-band activity (solid lines) and without alpha (dashed lines). The three graphs on the right show amplitudes of the P1 component plotted as a function of pre-stimulus alpha power after 8–14 Hz activity was removed.

Amplitude values of the P1 ERP component plotted as a function of binned pre-stimulus alpha activity are illustrated in Figure 5A. The analysis revealed a relationship between pre-stimulus alpha power and the P1 component in controls, whereby greater pre-stimulus alpha led to greater P1 amplitudes for the 8% (F(1,418) = 14.86, *p = 0.0001*; R^2^ = .06), and 57% (F(1,418) = 28.54, *p = 1.51*×*10^−7^*; R^2^ = .09) contrast conditions and a trend towards significance for the 2% contrast condition (F(1,418) = 3.67, p = 0.056; R^2^ = .01). In patients, we did not observe an effect for the 2% (F(1,418) = 0.023, p = 0.631; R^2^ = .0001), 8% (F(1,418) = 2.73, p = 0.0987; R^2^ = .006*)* or 57% (F(1,418) = 2.57, p = 0.1091; R^2^ = .006) contrast conditions.

We further tested whether the relationship between pre-stimulus alpha and P1 amplitude was due to interactions with post-stimulus alpha activity during the P1 timeframe. To test this, the ERP data were bandstop filtered from 8–14 Hz on every trial and the same binning procedure as above was performed. This analysis still revealed a significant relationship between pre-stimulus alpha and P1 amplitudes for the 8% (F(1,418) = 6.62, *p = 0.01*; R^2^ = .04) and 57% (F(1,418) = 15.59, *p = 9.22*×*10^−5^*; R^2^ = .06) contrast levels, but no effect for the 2% contrast condition (F(1,418) = 1.84, p = 0.175; R^2^ = .004). These data are illustrated in Figure 5B. Taken together, these data indicate that background alpha may interact with other frequency bands to bring about enhancements in the P1 component.

### Correlations between Alpha-activity, Medication and Symptoms

Pearson correlation analysis did not show a significant relationship between any alpha-band measures and CPZ equivalents in patients (p>.2). The Brief Psychiatric Rating Scale (BPRS) total scores were negatively correlated with right-hemisphere ERD across each contrast condition (n = 18∶2%: r = −.48, *p = .044*; 8%: r = −.49, *p = .04*; 57%: r = −.53, *p = .024*).

## Discussion

This study focused on investigating background and event-related alpha activity in patients with schizophrenia and their relationship to stimulus processing. Patients with schizophrenia showed decreased activity in pre-stimulus alpha with no differences in the post-stimulus period, relative to controls, indicating that reductions in alpha ERD are largely driven by impairments in background alpha. Moreover, the data showed that pre-stimulus alpha modulated the amount of alpha desynchronization in both groups, suggesting that ERDs are not solely a function of gain-related mechanisms. In addition, the data revealed a positive linear relationship between pre-stimulus alpha amplitude and the P1 ERP response, but only in controls. Taken together, these findings reveal a severe dysfunction in patients’ alpha activity, which may be a contributing factor to sensory processing deficits in this population.

### Background and Event-related Alpha Activity in Patients with Schizophrenia

A consistent finding in the schizophrenia literature is a significant decrease in patients’ alpha ERD [47], [50], [51]. One interpretation is that ERD reductions reflect impairments in sensory gating and/or task-related inhibitory mechanisms, which are driven by reductions in post-stimulus alpha activity relative to the pre-stimulus period. However, an alternative view is that ERD reductions arise, at least in part, from deficits in background alpha. Our results align with the latter assertion. Specifically, we found a substantial drop in patients’ pre-stimulus alpha, that was accompanied by normal post-stimulus alpha activity, indicating that ERD deficits in schizophrenia arise from a decrease in the range over which alpha desynchronizes before reaching floor levels. Further, our dataset showed that ERD reductions stemming from low levels in background alpha are not exclusive to patients, but are also seen in controls’ ERDs. The data revealed substantially lower ERDs in controls when their pre-stimulus alpha levels were lower than patients. These findings indicate that alpha desynchronization is dictated, at least in part, by the range over which background alpha operates.

Patients’ deficits in background alpha were observed in both hemispheres. However, patients showed a trend towards greater pre-stimulus alpha over the left compared to the right hemisphere, while healthy controls displayed the opposite pattern. Asymmetric hemispheric abnormalities have been previously reported in patients with schizophrenia [69], [70], [71], [72]. It is possible that alpha asymmetries observed in this study are driven by lateralized effects in parietal regions, which embody a fraction of the neural nodes that index different forms of attention [70]. However, it is also possible that these right-hemispheric biases could be driven by activations of the right fusiform cortex in response (or anticipation) to facial stimuli [73], [74].

A further goal of this study was to determine whether phasic-alpha modulations in patients are related to magnocellular dysfunction in schizophrenia. The magnocellular visual pathway responds to low contrast and shows a steeply rising response to low-to-mid contrast levels, after which the response plateaus at higher contrast (nonlinear contrast gain mechanism; [75]). The parvocellular pathway does not respond until ∼10% contrast [76]. This nonlinear pattern was not observed in the phasic-alpha response to increasing contrast, indicating that phasic-alpha oscillations are not subserved by nonlinear visual gain mechanisms.

### Relationship between Pre-stimulus Alpha and Sensory Processing

An important follow-up question on the background alpha findings, is whether pre-stimulus alpha holds strong bearings to stimulus processing, a relationship that may further our understanding of sensory processing deficits in patients with schizophrenia. Correlations between pre-stimulus alpha and stimulus processing have been previously established. For example, the phase of pre-stimulus alpha modulates the amplitude of the visual P1 [17], [38], [39], [40] and auditory N1 ERP components [77] in control populations. Other studies have shown relationships between pre-stimulus alpha power and ERP activity, whereby participants that elicit higher pre-stimulus alpha tend to exhibit larger amplitudes of the visual P300 ERP component [19], [41], while a separate study revealed an inverse relationship between pre-stimulus alpha power and the auditory P300 ERP component [20]. Here, we showed that pre-stimulus alpha power reliably predicted the amplitude of the visual P1 component at the single-trial level, but only in controls. This effect highlights the strong affinity between transient phase-locked post-synaptic potentials and ongoing fluctuations in background alpha. This is significant because it indicates that baseline alpha-oscillatory activity plays a fundamental role in shaping the responses of neural ensembles at early stages of sensory processing, a mechanism that may be flawed in schizophrenia patients. We posit that the functional role of this background alpha mechanism is to decrease the ‘noise’ in early sensory areas, such that incoming signals are processed with greater precision (i.e. increase the signal to noise ratio - SNR). Further, while speculative, the relationship between pre-stimulus alpha and the P1 response may not only index suppression of background noise, but may also reflect reductions in ‘correlated noise’ between cells [78], a hypothesis that merits further investigation.

We showed that the positive relationship between pre-stimulus alpha power and P1 amplitude was not solely a byproduct of a concomitant increase in alpha during the post-stimulus timeframe. Band-stop filtering the ERP data from 8–14 Hz revealed that pre-stimulus alpha was still a reliable predictor of the P1 amplitude. Although this relationship was statistically significant, the degree of correlation was considerably diminished. The fact that this relationship survived the band-stop filtering procedure invites the proposition that background alpha may interact with other frequency bands to bring about enhancements in the P1 component. A possible candidate is oscillatory activity in the gamma-band (>30 Hz) since patients with schizophrenia exhibit severe deficits in both these rhythms (see [51]), but also in their interactive nature [79].

### Limitations of the Study

Several caveats are important to note. First, pre-stimulus alpha and P1 amplitude are both quite low in patients, particularly at the 2% contrast level. Therefore, the lack of correlation between these two indices in patients may reflect reductions in SNR, which in turn may have hindered the likelihood of observing a correlation with our methods. Second, our task required little effortful attention. Thus, further work is necessary to determine whether the background alpha deficits as well as the pre-stimulus alpha/P1 relationship are similarly affected in paradigms that involve more effortful and demanding cognitive states. Third, all patients were receiving medication at the time of testing.
